# Pathways from women's group-based programs to nutrition change in South Asia: A conceptual framework and literature review

**DOI:** 10.1016/j.gfs.2017.11.002

**Published:** 2018-06

**Authors:** Neha Kumar, Samuel Scott, Purnima Menon, Samyuktha Kannan, Kenda Cunningham, Parul Tyagi, Gargi Wable, Kalyani Raghunathan, Agnes Quisumbing

**Affiliations:** a International Food Policy Research Institute, Poverty Health and Nutrition Division, Washington DC, USA; b Helen Keller International, Kathmandu, Nepal; c University of Glasgow, Scotland, UK; d Program in International Nutrition, Division of Nutritional Sciences, Cornell University, Ithaca, New York, USA

## Abstract

Improving the nutritional status of women and children in South Asia remains a high public health and development priority. Women's groups are emerging as platforms for delivering health- and nutrition-oriented programs and addressing gender and livelihoods challenges. We propose a framework outlining pathways through which women's group participation may facilitate improvements in nutrition. Evidence is summarized from 36 studies reporting on 24 nutritional indicators across infant and young child feeding (IYCF) practices, intake/diet, and anthropometry. Our findings suggest that women's group-based programs explicitly triggering behavior change pathways are most successful in improving nutrition outcomes, with strongest evidence for IYCF practices. Future investigators should link process and impact evaluations to better understand the pathways from women's group participation to nutritional impact.

## Highlights

• Women’s group programs in South Asia are starting to promote health and nutrition.
• Achieving nutritional impacts is complex and operates through multiple pathways.
• We outline income, agriculture, health behavior change, and rights pathways.
• Evidence is strongest for programs triggering the health behavior change pathway.

## 1 Introduction

Despite a large mortality rate reduction over the past twenty-five years, poor maternal and child nutrition continue to plague South Asia (Akseer et al., 2017). In most South Asian countries, one in three children under five years of age are stunted and eighteen percent (Nepal) to thirty-six percent (India) of women of reproductive age (15–49 years) are underweight (Akseer et al., 2017). Micronutrient deficiencies are also highly prevalent in the region. Anemia affects 54% of children aged 6–59 months and 42% of women aged 15–49 years in the South-East Asia region (World Health Organization, 2015), and 44–50% of preschool children in South Asia have severe vitamin A deficiency (Akhtar et al., 2013). Furthermore, the use of key nutrition-promoting practices during the 1000-day period between conception and a child's second birthday remains low, with only about half of South Asian women attending four antenatal care visits, initiating breastfeeding (BF) within one hour of birth, or practicing exclusive BF for the first 6 months (Akseer et al., 2017). Poor nutrition during this critical developmental period can lead to physiological, cognitive, and social shortcomings later in life, perpetuating the intergenerational cycle of poor human development (Crookston et al., 2013, Lozoff et al., 2013). The engagement of women and communities is fundamental to breaking this cycle (Rosato et al., 2008).

Women are central actors in achieving better household nutrition (Nisbett et al., 2017). Aside from being child bearers and caregivers with a more direct influence on fetal and infant health, compared to men, women choose to allocate more resources toward their family's health and nutrition (World Bank, 2012). However, given persistent gender inequalities in many developing countries, women often lack the autonomy and decision-making power within the household to make key decisions leading to better health and nutritional outcomes (Cunningham et al., 2014), and the resources with which to implement those decisions. Bringing women together in groups where they can share their experiences, gain access to resources and build knowledge, skills, and social networks is increasingly recognized as a potential strategy to empower women (Brody et al., 2015) and may also be a way to improve maternal and child health and nutrition.

Women's groups vary in size, generally ranging from 2 to 24 women, who meet regularly to discuss issues related to work, income, credit, and savings, agricultural livelihoods, health, or gender (Fottrell et al., 2013, More et al., 2012, Morrison et al., 2010). These groups are growing in salience as a platform used both by governments and non-governmental organizations for implementing development interventions in South Asia and worldwide. For example, in India alone, the government's flagship poverty alleviation program, the National Rural Livelihoods Mission has organized 33 million households into women's groups since 2011 (National Rural Livelihoods Mission, 2017). Non-governmental organizations across Asia and Africa are also mobilizing women's groups, with some encouraging federation of groups into self-governing and self-sustaining entities.

Large gaps remain in the literature linking participation in women's groups to health outcomes (O’Malley and Burke, 2016). Existing work evaluating impacts of women's group programs has focused on income-related activities (Buvinić and Furst-Nichols, 2016), social capital (Moestue et al., 2007), and participation of women in political processes (DHAN Foundation, 2016). In the context of health, studies have shown significant impacts on newborn health (Biscaye et al., 2014, Prost, 2013). In 2014, the World Health Organization strongly recommended community mobilization through facilitated participatory women's groups to improve newborn health (WHO, 2014), and a recent commentary called for further scaling up of these groups (Paul, 2016). The feasibility and effectiveness of integration of nutrition-focused interventions into existing activities undertaken by such groups has not yet been widely tested.

A previous report synthesized evidence on the effectiveness of women's groups on health, finance, agriculture, and empowerment objectives in both South Asia and Africa (Biscaye et al., 2014). Across the studies reviewed in this report and other papers (Brody et al., 2015) are insights on the diversity of women's group programs. The heterogeneity of group-based programs is expected, in turn, to lead to different pathways to impact on diverse outcomes. The development of a framework for agriculture-nutrition linkages that recognizes different pathways to impact has strengthened and consolidated research and policy efforts in that arena (Kadiyala et al., 2014). Although existing frameworks (Saville et al., 2016; Nair et al., 2015) link participatory approaches in behavior change interventions to health and nutrition outcomes, a more comprehensive framework to guide the thinking around women's group programs, more broadly defined, and potential pathways to impact does not yet exist, but would be instrumental to interpreting and framing the available evidence.

With this gap in mind, we propose a conceptual framework linking women's group-based programs to nutritional outcomes through hypothesized causal pathways. We also review and synthesize the evidence on the impacts of women's group programs and women's group membership on maternal and child nutrition outcomes in South Asia, specifically to map the available evidence to the conceptual framework. Finally, we examine the relative “success rate” of different group-based strategies in improving nutrition by summarizing findings across group program typologies and pathways.

In remaining sections of the paper, we first describe the conceptual framework (Section 2), describe methods for the literature review (Section 3), summarize evidence relating women's group programs to nutrition outcomes (Section 4), map the evidence onto our proposed conceptual framework (Section 5), and discuss our findings (Section 6).

## 2 Conceptual framework

### 2.1 Approach

We drew on three sources of information to guide the formation of a conceptual framework linking women's group programs to nutritional outcomes: 1) the framework for determinants of nutritional outcomes from the 2013 Maternal and Child Nutrition series published in *The Lancet* (Black et al., 2013), 2) the Tackling the Agriculture-Nutrition disconnect in India initiative's mapping of evidence along pathways linking agriculture and nutrition (Kadiyala et al., 2014) and 3) a typology of women's group programs and the potential inputs and activities associated with these programs, based on our own experience and knowledge. In this framework, we focus on dietary quality at the household level and among children and women, infant and young child feeding (IYCF) practices and anthropometric outcomes for women and children.

### 2.2 Pathways to nutrition impact

**Fig. 1:**
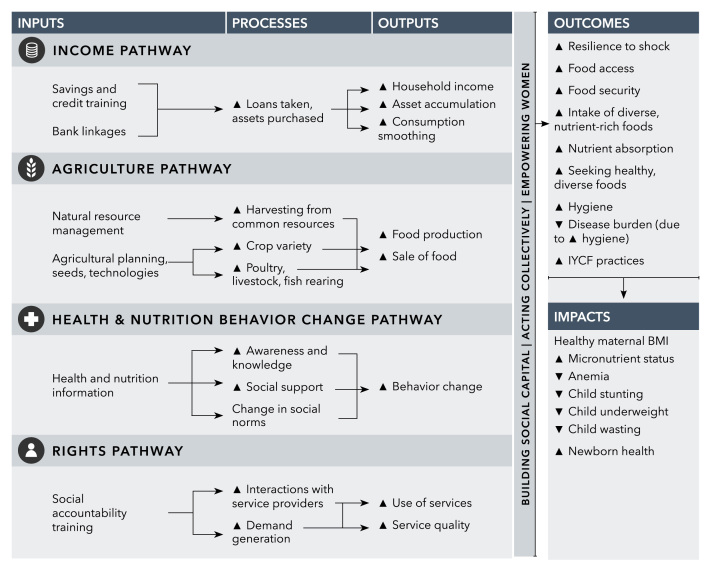
Conceptual pathways from women's groups to maternal and child nutrition.

We envision four distinct pathways from women's group-based programs to improved nutrition: 1) generation of income through savings and credit activities to improve purchasing power, 2) engagement of women to improve agricultural livelihoods, 3) behavior change communication (BCC) to improve health and nutrition awareness and knowledge, and 4) building social accountability and community demand for government programs that may improve nutritional outcomes (Fig. 1). Building social capital, acting collectively, and promoting women's empowerment are core components of each pathway that enable achievement of impacts (Box 1). There are potentially other intermediary steps in the pathways including feedback/mutually reinforcing loops. However, to make the framework generalizable to different program agendas as well as multiple outcome and impact pathways, we chose 1) not to list the many intermediary steps and 2) not to include arrows from outputs to outcomes or impacts. Below, we discuss each pathway and potential routes to impact.

Box 1 **Cross-cutting themes that promote achieving nutrition impact through women's group programs.**

*Building social capital*

Women's groups help to create a comfortable space where women can voice their opinions and share experiences with fellow members. When a group is initially formed, exercises may be conducted to teach listening skills and build trust. Likewise, group meetings may begin with songs or games to facilitate participation by all members. Groups that do not emphasize activities to create group cohesiveness, such as those rolled out by governments, may not be as effective in building social capital. Aside from the “social” aspects of social capital, groups have also been used as platforms for service delivery, and, working through group-based lending schemes, have acted as collateral substitutes. In effect, social capital helps to build other forms of capital.

*Acting collectively*

Some group-based programs emphasize collective action. For example, participatory learning and action (PLA) strategies enable and engage group members to identify shared problems, plan strategies, act together, and assess impacts (Harris-Fry et al., 2016, Kanani et al., 2015). While PLA approaches also emphasize group mobilization, confidence, and trust-building, the emphasis on problem solving, planning, and collective action distinguishes this approach from those that simply transmit information to members, but do not necessarily build community capacity to act collectively. Thus, although individual group members are the direct target of inputs along the pathways to nutrition impact, the effects are amplified and broadcasted when the community takes action collectively (Desai and Joshi, 2013). For example, changing social norms that have implications for nutritional wellbeing, requires actors at multiple levels—from husbands to village leaders—to be involved. In some PLA interventions, representatives from different women's groups within or across villages can gather to plan community workshops and awareness campaigns that help build skills and spread knowledge to non-members.

*Promoting women's empowerment*

Reducing the gender gap has received much global attention in the public health domain and is integral to most women's group strategies. To benefit from any inputs—income, agriculture, health and nutrition BCC, or social accountability training—women need to have some level of control or agency over their own decisions and be respected within their communities. Promoted strategies may include women's financial independence, running for elected positions in political offices or village councils, sharing of household chores by men and women, building perceptions of autonomy and self-wellbeing, negotiating skills with husbands, and control over reproductive choices, among others. As the disempowered woman builds confidence and gains support from men and the community at large, she will be better able to make decisions that promote her own health and that of her children (Sraboni et al., 2014).

#### 2.2.1 Generation of income through savings and credit activities

Participation of women in savings and credit or joint-liability groups where members pool financial resources and can take small loans, or in groups that create linkages with external lenders such as banks, is hypothesized to lead to increased household income, asset accumulation, and the ability to ensure food and nonfood consumption against adverse shocks (Banerjee, 2013). Both agricultural and non-agricultural assets can generate income streams which, in turn, could be spent on items that directly or indirectly enhance nutrition as well as healthcare or education, which have multiple long-term nutrition implications (Kadiyala et al., 2014).

#### 2.2.2 Engagement of women in agricultural livelihoods

Women play a key role in achieving food security as agricultural producers, income-earners, and caregivers, making up 43% of the agricultural workforce (35% in South Asia) (FAO, 2011). Women's greater control of agriculture-derived income is hypothesized to benefit households’ nutrition, given evidence that resources controlled by women tend to improve households’ health and nutrition outcomes (World Bank, 2012). Improved agricultural practices and management of natural resources could also mitigate fluctuations in year-round food availability in contexts with seasonal variability. Women's groups have been used to deliver agricultural inputs such as farming technologies and small-scale irrigation that improve productivity, and are a platform for involving women in agricultural planning (Vir and Koehler, 2017). Training women to have a greater role in agriculture would be expected to lead to changes in agricultural practices at household and community levels such as growing more diverse crops or using water-saving irrigation techniques. The number of groups to which women belong has been shown to be positively associated with some measures of household food security in Bangladesh, such as household calorie availability and dietary diversity (Sraboni et al., 2014). Such groups are often viewed as loci for the formation of social capital, which, especially for poor women, can substitute for and facilitate the building of other forms of capital (Kumar and Quisumbing, 2011).

#### 2.2.3 Behavior change communication to improve health and nutrition awareness and knowledge

Provision of health and nutrition information to women in a group setting by trained community workers has shown clear impacts on newborn mortality (Prost, 2013). In the context of behaviors that are highly dependent on social and cultural norms, group-based strategies may specifically facilitate peer-to-peer exchange that encourages behavioral change through reshaping of norms. Nutrition behavior change topics in the context of women's groups include dietary diversity, IYCF practices, sanitation and hygiene, and access to and utilization of health services. Promoting healthy behaviors and health care-seeking among women through group-based strategies is hypothesized to improve knowledge, motivation and social support for these behaviors and, in turn, lead to better household practices around diet, health, and hygiene, and ultimately better maternal and child nutrition outcomes.

#### 2.2.4 Building social accountability and community demand for government schemes

Poor women may often not know what public health and social services/programs are available to them, and what they are entitled to as beneficiaries of these programs (Brody et al., 2015). Training and supporting women and other members of poor communities to hold service providers accountable for delivering high quality and timely services offers important opportunities for achieving better nutrition outcomes via women's groups. As women learn about their rights and get linked to services through group-based programs, service use and quality are expected to increase (Brody et al., 2015).

## 3 Literature review methodology

### 3.1 Search strategy

We searched databases for peer-reviewed journal articles and websites for grey literature to identify evidence for the impact of participation in women's groups on nutrition outcomes. Search terms are shown in Table 1. Only papers and reports published in English between 1980 and July 2016 were considered, and two additional papers, one from 2016 and one from 2017, were identified and added during the peer review process in November 2017. To ensure that we did not miss other relevant studies, we conducted a search for other studies that may have come between July 2016 and November 2017. But the search did not return any studies that fit our search criteria. Databases included Embase, Global Health, Eco Lit, IBSS, Web of Science, Medline, Scopus and WorldCat. Websites included government websites (particularly Ministries of Agriculture, Women and Child Development, and Health and Family Welfare), and websites of research institutions, multilateral institutions, and United Nations agencies (World Bank, Consultative Group on International Agriculture Research, UN System Standing Committee on Nutrition, Food and Agriculture Organization of the UN, UN Women). Although we followed systematic review search protocols, our review is, strictly speaking, not a systematic review but a literature review that was informed by a systematic search process.

**Table 1:** Search terms used for database searches.

**Women**		**Platform**		**Nutrition (Impact)**		**Location**
Wom*	AND	Group*	AND	Nutri*	AND	South* Asia*
Moth*		Collectiv*		Micronutri*		Afghanistan*
Mat*		Organi*		Macronutri*		Bangladesh*
		Coop*		Body Mass Index		Bhutan*
				Anthropometr*		India*
				Arm circumferen*		Maldives*
				Stunt*		Nepal*
				Wast*		Pakistan*
				Underweight		Sri Lanka*
				Anemi*		
				Hemoglobin		
				Diet*		
				Food*		
				Feed*		
				Calori*		
				Grow*		
				Breast*fe*		
				Complementar* Feed*		
				Birth* weigh*		
				Vitamin*		
				Mineral*		

Within a column, OR terms were used.

### 3.2 Eligibility criteria

Only studies done in South Asian countries—Afghanistan, Bangladesh, Bhutan, India, Maldives, Nepal, Pakistan, Sri Lanka—were considered. Study subjects had to include members of a women's group or collective, or children of group members, and nutritional outcomes had to be measured quantitatively. Nutritional outcomes considered included anthropometry, IYCF practices, micronutrient and macronutrient intake, dietary diversity, and vitamin and mineral deficiencies at the child, maternal, or household level. Nutrition knowledge/awareness and decision-making on nutrition were not considered as outcomes. Literature reviews were ineligible but were screened for additional relevant articles.

### 3.3 Article screening and data extraction process

**Fig. 2:**
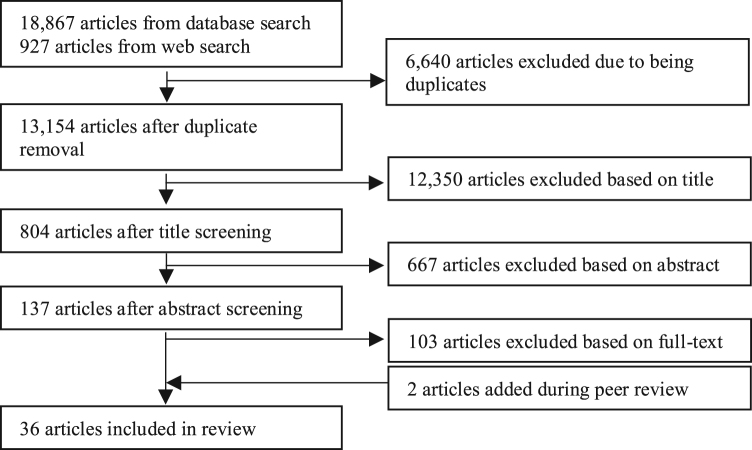
Article screening process.

A pooled list of articles and reports from the systematic search was created and duplicates were removed. Titles, abstracts, and full text of the articles were independently screened for relevance by two independent reviewers, with a third reviewer to resolve discrepancies (Fig. 2). Additional articles from literature reviews, references in included articles, from authors' own knowledge and during the peer review process were added and subjected to the same screening process. Data were extracted from the included articles for study design, year, country and context, women's group category, program details, age of the women's group members and/or their children, outcomes measured, statistical approach, findings and significance, covariates included, and strengths and limitations. Pilot data extraction was conducted independently by two reviewers for five articles and, after discussion among the group to resolve differences, a single reviewer extracted data for the remaining articles. After data extraction was completed for all included articles, another team member conducted accuracy checks by going back to each full text article.

### 3.4 Mapping reviewed programs onto conceptualized nutrition impact pathways and summarizing findings across program types and pathways triggered

The studies included in this review consist of a range of women's group types with varied program inputs. Program inputs for each study were examined in-depth to determine which conceptual pathways to nutrition impact were directly triggered and which were potentially or indirectly triggered. Because of our interest in which program types were generally most successful in achieving nutrition impacts and whether layering interventions on specific group types was related to success, we tabulated the number of studies and nutrition outcomes reported both by group type and by impact pathway triggered. The same outcome indicator reported for different subgroups, for example younger and older children, was counted as more than one measure. Finally, as a quality assessment, we tabulated findings by study design to assess the range of study types across which certain impacts were seen. For each of these tabulations—by group type (Table 6), impact pathway triggered (Table 7), and study design (Table 4)—we report the total number of studies and outcomes across those studies, as well as the number that were reported as significantly (*p* ≤ 0.05) positively associated with the program (i.e. nutrition was improved because of the program inputs delivered through a women's group or because of participation in a women's group), negatively associated with the program (i.e. the program had a detrimental effect on the nutrition measure), or non-significant (*p* > 0.05). No statistical tests were run as part of this review; the determination of significance (or lack thereof) of all findings was as reported by the reviewed studies, which all used a variety of statistical tests.

## 4 Summary of evidence relating women's group programs to nutrition outcomes

### 4.1 Overview of selected studies

Our search process yielded 36 eligible articles and reports published over twenty-four years and spanning four countries: India (18 studies), Bangladesh (13 studies), Nepal (4 studies) and Pakistan (1 study) (Table 2). Journal articles (29 studies), grey literature (6 studies), and one book chapter were included. A wide range of programs delivered via different group types including microfinance groups (5 studies), livelihoods self-help groups (SHGs) (6 studies), multi-sectoral groups (4 studies), and BCC groups (18 studies), with three studies including multiple group types or not reporting a specific group type. Where programs were delivered through women's groups, it was often impossible to disentangle the separate effect of the program from that of group membership. Although our emphasis was on final outcomes (we did not include studies that only reported on intermediate outcomes), thirteen of the included studies also reported findings on intermediate outcomes, such as per capita income, empowerment, and production diversity, which help explain how or why nutrition impacts were or were not achieved.

**Table 2:** Summary of studies examining effects of programs targeted at women's groups on nutrition outcomes in women and children (in descending order of year of publication).

**Ref.**^a^	**Country**	**Study design**	**Group targeted**^b^	**Intervention description**	**Intermediate outcomes reported**^c^	**Nutritional outcomes reported**
(Nair et al., 2017)	IN	RCT	BCC group	BCC through a facilitated PLA cycle, home visits during pregnancy and early childhood	None	BMI, MUAC, nutrition wellbeing, diet diversity (woman); birthweight, HAZ, WHZ, WAZ, MUAC, stunting, wasting, underweight, exclusive BF, CF introduction, protein intake, diet diversity, minimum meal frequency (child)
(Harris-Fry et al., 2016)	BG	Quasi-experimental	BCC group	BCC through a facilitated PLA cycle	None	Diet diversity (woman)
(Tripathy et al., 2016)	IN	RCT	BCC group	BCC through a facilitated PLA cycle	None	Early BF initiation, exclusive BF
(Acharya et al., 2015)	IN	RCT	BCC group	BCC delivered by health worker on maternal and newborn health	None	Feeding colostrum, early BF initiation, exclusive BF
(Malapit et al., 2015)	IN	Cross-sectional	Multiple/unspecified group	NA; only measured impact of group membership	Empowerment, production diversity	BMI, HAZ, WAZ, WHZ, diet diversity (woman)
(Younes et al., 2015)	BG	Quasi-experimental	BCC group	BCC through a facilitated PLA cycle	None	Exclusive BF, diet diversity (child)
(Habib and Jubb, 2015)	BG	Cross-sectional	Microfinance group	NA; only measured impact of group membership	Income	Meals consumed per day (woman)
(Saha et al., 2015)	IN	Quasi-experimental	Multi-sectoral group	Health care/education programs layered on microfinance programs	None	Feeding colostrum
(Johnson et al., 2014)	IN	Cohort with pre/post evaluation	Multi-sectoral group	BCC on maternal and child health	Economic wellbeing, social capital	Early BF initiation, timely introduction of complementary foods
(Prennushi and Gupta, 2014)	IN	Quasi-experimental	Livelihoods self-help group	Group formation, federation building, savings promotion, linkages to banks and government schemes	Expenditure, beneficiary of government schemes, child education, empowerment	Exclusive BF
(Miller et al., 2014)	NP	RCT	Livelihoods self-help group	Livestock distribution and related training	Socioeconomic status, income, production diversity	HAZ, WAZ, WHZ, MUACZ
(Houweling et al., 2013)	IN	RCT	BCC group	BCC through a facilitated PLA cycle	None	Early BF initiation
(Saha et al., 2013)	IN	Cross-sectional	Multiple/unspecified group	NA; related presence of SHG in village to outcomes in cross-sectional survey	None	Feeding colostrum
(Fottrell et al., 2013)	BG	RCT	BCC group	BCC through a facilitated PLA cycle plus health system strengthening	None	Early BF initiation, exclusive BF
(Roy et al., 2013)	IN	RCT	BCC group	BCC through a facilitated PLA cycle; additional emphasis on thermal care of newborn	None	Early BF initiation, exclusive BF
(Jalal and Frongillo, 2013)	BG	Quasi-experimental	Microfinance group	Provision of income earning assets, income allowance, training on entrepreneurial skills, strengthening sociopolitical livelihoods	None	BMI, HAZ, WAZ
(Deininger and Liu, 2013a)	IN	Quasi-experimental	Livelihoods self-help group	Group formation, federation building, savings promotion, linkages to banks and government schemes	Social capital, economic empowerment, political participation	Per capita daily energy and protein intake
(Deininger and Liu, 2013b)	IN	Quasi-experimental	Livelihoods self-help group	Group formation, federation building, savings promotion, linkages to banks and government schemes	Consumption expenditure, non-financial assets	Per capita daily energy and protein intake
(More et al., 2012)	IN	RCT	BCC group	BCC through a facilitated PLA cycle; focus on perinatal care	None	Early BF initiation, exclusive BF
(Kumar and Quisumbing, 2011)	BG	Quasi-experimental	Livelihoods self-help group	Training on improved vegetable production, polyculture fishpond production, microcredit provision	Food and nonfood expenditure, value of assets (including livestock), per capita income	Calorie, protein, vitamin A, and iron consumption (woman and child); HAZ, BMIZ, BMI, hemoglobin, stunting, wasting, low BMI, low hemoglobin
(Bhutta et al., 2011)	PK	RCT	BCC group	Health committee creation, birth attendant linkages, BCC on perinatal care for mother/newborn	None	Colostrum feeding, early BF initiation
(De and Sarker, 2011)	IN	Quasi-experimental	Microfinance group	Group formation, savings promotion, linkages to banks and government schemes, skills training	Empowerment	Protein intake (household), WAZ
(Azad et al., 2010)	BG	RCT	BCC group	BCC through a facilitated PLA cycle plus health system strengthening	None	Exclusive BF
(Tripathy et al., 2010)	IN	RCT	BCC group	BCC through a facilitated (by community volunteer) PLA cycle, health committee formation	None	Early BF initiation, exclusive BF
(Kumar et al., 2008)	IN	RCT	BCC group	BCC on essential newborn care plus sticker that indicates hypothermia (ThermoSpot)	None	Early BF initiation, given prelacteal
(White and Masset, 2007)	BG	Quasi-experimental	BCC group	Community-based nutrition counseling, child growth monitoring, supplementary feeding for malnourished children	None	Weight gain during pregnancy
(Hallman et al., 2007)	BG	Cohort study with mixed methods	Livelihoods self-help group	Training on improved vegetable production, polyculture fishpond production, microcredit provision	Household expenditure, income, empowerment, collective action	WHZ
(Eklund et al., 2007)	NP	Repeated cross-sectional surveys	Multi-sectoral group	Intensive: child growth monitoring, group empowerment, funds for community initiatives	Group maturity	Stunting, underweight
				Moderate: savings promotion, bank linkages		

(Galab et al., 2006)	IN	Cross-sectional retrospective analysis	Multiple/unspecified group	NA; analysis of whether self-reported active group membership in previous 12 months predicts outcomes	Social capital	HAZ
(Wade et al., 2006)	NP	RCT	BCC group	BCC with focus on perinatal care	None	Feeding colostrum
(World Bank OED, 2005)	BG	Quasi-experimental	BCC group	Community-based nutrition counseling, child growth monitoring, supplementary feeding for malnourished children	None	HAZ, WAZ, WHZ
(Manandhar et al., 2004)	NP	RCT	BCC group	BCC with facilitated PLA cycle, focus on perinatal care	None	Feeding colostrum, early BF initiation
(Khatun et al., 2004)	BG	Cohort study	Multi-sectoral group	BRAC: microcredit, female education/health services; ICDDR,B: home visits, infection and malnutrition treatment, family planning	None	Stunting
(Pitt et al., 2003)	BG	Quasi-experimental	Microfinance group	Microcredit provision	None	MUAC, height, BMI
(Pitt and Khandker, 1996)	BG	Quasi-experimental	Microfinance group	Microcredit provision	Household expenditure, labor supply, women's non-land assets, schooling, contraceptive use, fertility	BMI
(Lal et al., 1992)	IN	Cohort study	BCC group	Participatory health communication by community workers	None	Early BF initiation, timely introduction of complementary foods

*Alphabetized list of abbreviations*: BCC, behavior change communication; BF, breastfeeding; BG, Bangladesh; BMI, body mass index; BMIZ, body mass index Z-score; BRAC, building resources across communities; HAZ, height-for-age Z-score; ICDDR,B, international center for diarrhoeal disease research, Bangladesh; IN, India; MUAC, mid-upper arm circumference; MUACZ, mid-upper arm circumference Z-score; NA, not applicable; NP, Nepal; PK, Pakistan; PLA, participatory learning and action; RCT, randomized controlled trial; SHG, self-help group; WAZ, weight-for-age Z-score; WHZ, weight-for-height Z-score.

a References are shown in descending order of year.

b See Box 2 for descriptions of group types.

c We define intermediate outcomes as non-nutrition outcomes that may lead to nutrition impacts.

Study designs included cross-sectional, mixed-method, pre-post program evaluation, quasi-experimental, and randomized controlled trials (RCTs) (Table 2, Table 3). Studies on IYCF practices were mostly RCTs, whereas those reporting on diet or anthropometric outcomes were largely quasi experimental or cross-sectional studies. A total of 167 findings for 24 unique nutrition outcomes were reported (Table 3) with positive/significant, negative/significant, and non-significant findings for 40 (24%), 11 (7%), and 116 (69%) outcomes, respectively. These 167 findings are spread across the three broad outcome categories: intake and diet, IYCF practices, and anthropometry. Our unit of analysis or observation is the unique pair represented by the study and a particular outcome for a given sub-group; we have 167 such unique pairs. Each study included in our review could report on multiple outcomes and thus a given study could have a positive significant result, a negative significant result as well as a null result for different outcomes or the same outcomes but defined for different sub-groups. For example, 19 studies reported on IYCF practices and, among these, 12 studies reported positive significant results, 1 reported a negative significant effect and 10 reported null effects (12 + 1 + 10 = 23 > 19 because of multiple outcomes and multiple findings across subgroups per outcome reported by a given study).

**Table 3:** Findings on maternal and child nutrition outcomes across the three outcome categories.

Outcome	Total studies (outcomes)	Pos./Sig. studies (outcomes)*	Neg./Sig. studies (outcomes)*	Null/Non-sig. studies (outcomes)*
**Intake and diet**	9 (51)	4 (5)	2 (6)	6 (40)
Diet diversity	4 (6)	0 (0)	1 (1)	3 (5)
Minimum meal frequency	1 (1)	0 (0)	0 (0)	1 (1)
Nutrient intake-energy/calories	3 (10)	1 (1)	1 (3)	2 (6)
Nutrient intake-iron	1 (8)	0 (0)	0 (0)	1 (8)
Nutrient intake-protein	5 (12)	2 (2)	1 (2)	3 (8)
Nutrient intake-vitamin A	1 (8)	1 (1)	0 (0)	1 (7)
Nutrient status-Hb/anemia	1 (4)	0 (0)	0 (0)	1 (4)
Nutritional wellbeing (general)	2 (2)	1 (1)	0 (0)	1 (1)
**Infant and young child feeding practices**	19 (40)	12 (23)	1 (1)	10 (16)
Timing of complementary feeding	2 (2)	1 (1)	0 (0)	1 (1)
Colostrum feeding	6 (9)	5 (7)	0 (0)	2 (2)
Exclusive breastfeeding	10 (13)	4 (7)	0 (0)	6 (6)
Initiation of breastfeeding	12 (14)	7 (7)	0 (0)	5 (7)
Use of pre-lacteal	2 (2)	1 (1)	1 (1)	0 (0)
**Anthropometry**	14 (76)	5 (12)	3 (4)	14 (60)
Body mass index	6 (19)	1 (2)	0 (0)	6 (17)
Birthweight	1 (1)	0 (0)	0 (0)	1 (1)
Height-for-age Z-score	8 (20)	2 (4)	1 (2)	7 (14)
Height	1 (2)	1 (2)	0 (0)	0 (0)
Mid-upper arm circumference	3 (6)	1 (2)	0 (0)	2 (4)
Stunting	3 (6)	0 (0)	1 (1)	3 (5)
Underweight	2 (2)	0 (0)	0 (0)	2 (2)
Weight-for-age Z-score	6 (7)	0 (0)	0 (0)	6 (7)
Weight-for-height Z-score	6 (11)	1 (1)	1 (1)	6 (9)
Wasting	1 (1)	0 (0)	0 (0)	1 (1)
Weight	1 (1)	1 (1)	0 (0)	0 (0)
**Total**	36 (167)	20 (40)	5 (11)	25 (116)

Numbers in parentheses indicate the number of times each outcome was reported, which may be more than once in a given study if results were reported separately for different subgroups.

* Significance considered at p < 0.05 level; positive implies a benefit of intervention while negative implies the opposite; numbers indicate the number of studies finding positive and significant, negative and significant, or null (p > 0.05) effects. Some outcomes were reported more than once for subgroups, e.g. age groups, hence the greater number of outcomes compared to the number of studies.

**Table 4:** Findings on maternal and child nutrition outcomes by outcome category and study design.

Outcome category and study design	Total studies (outcomes)	Pos./Sig. studies (outcomes)*	Neg./Sig. studies (outcomes)*	Null/Non-sig. studies (outcomes)*
**Intake and diet**	9 (51)	4 (5)	2 (6)	6 (40)
Cross-sectional	2 (3)	1 (1)	0 (0)	1 (2)
Mixed methods	0 (0)	–	–	–
Pre-post program evaluation/study	0 (0)	–	–	–
Quasi experimental	5 (42)	3 (4)	2 (6)	3 (32)
RCT	2 (6)	0 (0)	0 (0)	2 (6)
**Infant and young child feeding practices**	19 (40)	12 (23)	1 (1)	10 (16)
Cross-sectional	1 (1)	1 (1)	0 (0)	0 (0)
Mixed methods	0 (0)	–	–	–
Pre-post program evaluation/study	2 (2)	2 (4)	0 (0)	0 (0)
Quasi experimental	3 (3)	2 (3)	0 (0)	1 (1)
RCT	13 (31)	7 (15)	1 (1)	9 (15)
**Anthropometry**	14 (76)	5 (12)	3 (4)	14 (60)
Cross-sectional	3 (8)	0 (0)	1 (1)	3 (7)
Mixed methods	1 (2)	0 (0)	1 (1)	1 (1)
Pre-post program evaluation/study	1 (3)	0 (0)	0 (0)	1 (3)
Quasi experimental	7 (44)	4 (10)	1 (2)	7 (32)
RCT	2 (19)	1 (2)	0 (0)	2 (17)

Numbers in parentheses indicate the number of times each outcome was reported, which may be more than once in a given study if results were reported separately for different subgroups.

* Significance considered at p < 0.05 level; positive implies a benefit of intervention while negative implies the opposite; numbers indicate the number of studies with outcomes found to be positive and significant, negative and significant, or null (p > 0.05).

### 4.2 Intake and diet outcomes

Nine studies shared findings on diet outcomes including energy, protein and carbohydrate intake, number of meals per day, and dietary diversity. These were a mix of RCT (1), quasi-experimental (6) and cross-sectional (2) studies. Overall, there was limited evidence to suggest that programs delivered via women's groups benefit diet outcomes.

A quasi-experimental study of improved vegetable and group fishponds interventions in Bangladesh had mixed results on dietary outcomes. Among the early female adopters of the vegetable technology that was disseminated through women's groups, vitamin A intakes improved but calorie and protein intakes declined (Kumar and Quisumbing, 2011). The group fishponds intervention led to no dietary improvements.

Participation in microfinance programs has had mixed impacts on dietary outcomes. In India, household protein intake was not associated with SHG membership in one study (De and Sarker, 2011) but was in another (Deininger and Liu, 2013a). In the latter, daily per capita consumption of calories and protein increased among the treatment group. In a subsequent study, the authors found that the benefit on energy and protein intake was higher with longer group participation and greater initial poverty (Deininger and Liu, 2013b). Likewise, in a study in Bangladesh, compared to members of a microfinance program who had participated for four or eight years, non-participants were less likely to consume three meals per day (Habib and Jubb, 2015).

Four studies reported on dietary diversity outcomes. Analysis of cross-sectional survey data showed that maternal and child dietary diversity were not related to group membership in Nepal (Malapit et al., 2015). A difference-in-difference analysis of a program involving health BCC through a facilitated PLA cycle in Bangladesh found greater dietary diversity improvement in the control compared to the treatment group (Younes et al., 2015). However, groups were not matched at baseline, with the control being worse off and thus having greater improvement potential. Another study reported that BCC delivered to women using a PLA approach improved women's dietary diversity (Harris-Fry et al., 2016). Finally, in a trial in India, BCC delivered through group meetings following a PLA approach and home visits resulted in a significant positive program effect on dietary diversity in woman and children as well as protein intake in children (Nair et al., 2017).

### 4.3 Infant and young child feeding behavior outcomes

Nineteen studies reported on IYCF practices, including practices such as timely introduction of complementary foods, colostrum feeding, exclusive breastfeeding, early initiation of breastfeeding, and use of pre-lacteals. Among the 19 studies assessing these practices, 13 were RCTs. Twelve of 19 found at least one positive and statistically significant outcome whereas 10 studies found at least one null effect (Table 3). Of 40 outcomes reported across these 19 studies, 23 (60%) were positive and significant.

Three articles reported on a 3-year cluster RCT in India, where facilitators engaged women's groups through a participatory learning and action (PLA) cycle (Houweling et al., 2013, Roy et al., 2013, Tripathy et al., 2010). No effect was found on early BF initiation but mothers who gave birth in clusters that received the program inputs were more likely to exclusively breastfeed their infants for ≥ 6 weeks compared to mothers in control clusters (Tripathy et al., 2010). The same group of researchers later conducted a RCT in India which involved a government health worker who performed home visits in addition to women's group meetings using the PLA approach; in this study, no intervention effects were seen for exclusive breastfeeding or timely introduction of complementary foods (Nair et al., 2017).

In a similar large cluster RCT in Nepal involving delivery of perinatal care information through facilitated PLA cycle, a positive treatment effect was found for not discarding colostrum, but not for early BF initiation (Manandhar et al., 2004). Group attendance appeared a modest determinant of success in another large program in Bangladesh. In the original trial, with a coverage of one women's group per 1414 population a small non-significant impact was found on exclusive BF (Azad et al., 2010). Increasing coverage 4–5-fold (one group per 309 population) was associated with an increase in exclusive BF and early initiation of BF (Fottrell et al., 2013).

Two large RCTs in India with similar program implemented in different contexts yielded different results. First, in rural villages, a package of essential newborn care delivered by community health workers at women's group meetings for 16 months (Kumar et al., 2008) led to lower use of pre-lacteal feeds and greater early BF initiation. Second, in a 3-year program targeted at urban slum dwellers, where a facilitator engaged women's groups on perinatal health through a PLA cycle (More et al., 2012), no impacts were seen on early BF initiation or exclusive BF. The authors attributed the lack of effects to low coverage, poor collective action, and failure to target the poorest slum dwellers.

In a RCT from Pakistan, both traditional birth attendants and lady health workers were trained in newborn care, and community health committees were formed to advocate for improved newborn care (Bhutta et al., 2011). BCC on these topics was delivered through women's group sessions over a 2-year period. Compared to women in control areas, those in treatment areas were more likely to feed colostrum and practice early BF initiation.

Three non-RCT studies in India provide moderate evidence for impacts on IYCF outcomes. A cross-sectional study found that, compared to those without an SHG, respondents from villages with an SHG were more likely to feed colostrum (Saha et al., 2013). An evaluation of the Indira Kranthi Patham program designed to empower women through livelihoods SHGs did not improve exclusive BF (Prennushi and Gupta, 2014). A 2-year BCC intervention delivered through women's groups improved early BF initiation and appropriate complementary feeding (Lal et al., 1992).

### 4.4 Anthropometric outcomes

Fourteen studies measured anthropometric outcomes including weight and body mass index (BMI) in women, and height, BMI mid-upper arm circumference (MUAC), weight for age z-score (WAZ), height/length for age z-score (HAZ/LAZ), weight for height z-score (WHZ), and stunting in children. Overall, few program or group membership effects were found. Five of the 14 studies found positive significant impacts of programs delivered through women's groups on anthropometric outcomes and 14 (18%) of 76 outcomes measured across those studies were significantly positively related to the program (Table 3). Most studies that did report positive significant impacts were quasi-experimental in design.

Studies using cross sectional data provided little evidence for an association between women's group membership and anthropometric outcomes. No association between group membership and child HAZ was found in India (Galab et al., 2006) and, in Nepal, the group membership component of the women's empowerment in agriculture index was not related to maternal or child anthropometry (Malapit et al., 2015). Across several studies in Bangladesh, participation in microfinance programs was not related to improved child HAZ, WHZ, or BMI (Jalal and Frongillo, 2013, Khatun et al., 2004, Pitt et al., 2003, Pitt and Khandker, 1996). One study compared NGO members to non-eligible non-members, thus the group membership effect on stunting was confounded by differences in socioeconomic status (Khatun et al., 2004). Another found impacts of credit delivered through women's groups on child MUAC and linear growth when credit was given directly to women and not men (Pitt et al., 2003).

Insights on the effects of program duration and intensity can be drawn from studies in three countries. Using propensity score matching, an evaluation of a large scale government nutrition program delivered via women's groups in Bangladesh found treatment effects on child HAZ, WAZ and WHZ after two years, which were not sustained after four years (White and Masset, 2007, World Bank OED, 2005). In India, being a member of a women's borrower group for ≥ 8 years was not, but amount saved through group savings activities was, associated with child WAZ (De and Sarker, 2011). More intense intervention delivery may not necessarily improve anthropometric outcomes. Compared to mothers’ groups receiving 1) intensive support involving child growth monitoring and small grants for community projects or 2) moderate support involving savings and credit promotion, stunting was lower in mature autonomous SHGs (Eklund et al., 2007), underscoring the importance of building effective leadership and management among the group members themselves.

Three programs focused on improving livelihoods through women's groups. Vegetable and fishpond interventions in Bangladesh were surveyed twice, in 1996–97 (Hallman et al., 2007) and in 2006–2007 (Kumar and Quisumbing, 2011). In the short term, the vegetable program groups experienced reduced child WHZ, while the fishpond intervention had a positive but insignificant impact. Impacts were sustained ten years later, as early adopters of the improved vegetable technology showed a reduction in the proportion of stunted girls and underweight boys, and an increase in women's BMI. The 2-year livestock promotion project in Nepal implemented through groups, and evaluated using a RCT found a treatment effect on child HAZ but not on other indicators (Miller et al., 2014).

In the recent BCC intervention in India involving a PLA approach and home visits, a small non-significant benefit of the program was seen on child length-for-age Z score at 18 months, but no effects were seen for any other anthropometry outcomes in either mothers or their children (Nair et al., 2017).

## 5 Mapping evidence onto conceptual framework of nutrition impact pathways

Although only 13 of 36 studies reviewed reported on intermediate outcomes, the pathway/s to impact triggered by each group-based program can be inferred based on information about the group type and program inputs. Program types and pathways to impact either explicitly or potentially triggered are shown in Table 5. Four major types of women's group-based programs emerged in the literature reviewed above: microfinance groups, livelihoods self-help groups (SHG), multi-sectoral groups, and BCC-oriented groups (Box 2). Three studies measured associations between group membership and nutritional outcomes across several programs and group types, which we categorized as “Multiple/Unspecified group based programs”.

**Table 5:** Nutrition impact pathways triggered by women's groups programs.

Intervention category and program name	Income Pathway	Rights pathway	Agriculture pathway	BCC+ pathway^a^	Studies
**Microfinance group-based interventions**					
BRAC-CFPR TUP	x	~	~		(Jalal and Frongillo, 2013)
Grameen/BRAC/BDRB	x	~	~		(Pitt et al., 2003, Pitt and Khandker, 1996)
ASA	x	~	~		(Habib and Jubb, 2015)
Several NGO/non-NGO programs	x	~	~		(De and Sarker, 2011)
**Livelihoods self-help group-based interventions**					
Indira Kranthi Patham (DPIP)	x	x	~		(Deininger and Liu, 2013a, Deininger and Liu, 2013b, Prennushi and Gupta, 2014)
Heifer International	x	x	x		(Miller et al., 2014)
Banchte Shekha	x	~	x		(Hallman et al., 2007, Kumar and Quisumbing, 2011)
**Multi-sectoral group-based interventions**					
SEWA/SKDRDP	x	x	x	x	(Saha et al., 2015)
Bandhan-Freedom from Hunger	x	~	~	x	(Johnson et al., 2014)
IWCDP/DPCP (Nepal)	x	~	~	x	(Eklund et al., 2007)
BRAC-ICDDRB	x	~	~	x	(Khatun et al., 2004)
**BCC group-based interventions**					
BINP				x	(White and Masset, 2007, World Bank OED, 2005)
BADAS				x	(Azad et al., 2010, Fottrell et al., 2013, Harris-Fry et al., 2016, Younes et al., 2015)
Sure Start				x	(Acharya et al., 2015)
Group mobilization through LHW				x	(Bhutta et al., 2011)
Group mobilization through ASHA				x	(Tripathy et al., 2016)
MIRA^c^				x	(Manandhar et al., 2004, Wade et al., 2006)
Ekjut^c^				x	(Houweling et al., 2013, Nair et al., 2017, Roy et al., 2013, Tripathy et al., 2010)
SNEHA^c^				x	(More et al., 2012)
ThermoSpot^c^				x	(Kumar et al., 2008)
Unnamed BCC intervention^c^				x	(Lal et al., 1992)
**Multiple/unspecified group-based interventions**^b^					
Multiple/unspecified	~	~	~	~	(Galab et al., 2006, Malapit et al., 2015, Saha et al., 2013)

x, explicitly triggered pathway given intervention design.

~, potentially triggered pathway given intervention design.

*Alphabetized list of abbreviations*: Ag., agriculture; ASA, association for social advancement; ASHA, accredited social health activist; BADAS, diabetic association of Bangladesh; BCC, behavior change communication; BDRB, Bangladesh rural development board; BINP, Bangladesh integrated nutrition program; BRAC-CFPR TIP, Bangladesh rural advancement committee-challenging the frontiers of poverty reduction-targeting ultra poor; BRAC-ICDDRB, Bangladesh rural advancement committee, international center for diarrhoeal disease research; DPIP, district poverty initiatives project; IWCDP/DPCP, integrated women and child development program/decentralized planning for child program; LHW, lady health worker; MIRA, mother and infant research activities; Nutr., nutrition; SEWA (SKDRDP), self-employed women's association (Shri Kshetra Dharmasthala Rural Development Project).

a In addition to behavior change communication, the intervention also included health and nutrition services including growth monitoring, supplementation, health checkups, and/or distribution of care packages.

b Includes programs involving multiple group types (e.g. both microfinance and livelihoods self-help groups) or did not specify which group type the program targeted.

c BCC only interventions, i.e. do not include other health and nutrition services.

Box 2 **Description of women's group types.**

*Microfinance-group*

Traditional microcredit programs are characterized by joint-liability women's groups that receive loans, income-building assets or grants from financial intermediaries. The primary pathway to nutrition impact in these programs is the income pathway*,* while indirect effects may occur through the agriculture and rights pathways as borrowers build productive and social capital.

*Livelihoods group*

Livelihoods-oriented groups are a model of microfinance popular in South Asia. Groups of women formed by governmental or non-governmental organizations practice internal saving and lending, and often engage in income-generating and development activities. Groups have more autonomy and are directly linked to the formal banking system unlike micro-finance groups. Other examples of livelihoods-oriented groups include homestead food production (HFP) programs as well as aquaculture or fishponds. These interventions influence nutrition through the income, agriculture and rights pathways.

*Multi-sectoral group*

Interventions that deliver a bundle of programs to simultaneously improve financial access, livelihoods, entitlements and health and nutrition in women. Savings and credit continue to remain the core activity of these groups. These programs improve nutrition through most, if not all impact pathways.

*Behavior change communication group*

Programs that deliver interventions focused solely on delivering information or reshaping/reinforcing social norms as a key pathway to improving health and nutrition of women and children. The groups of women formed as part of the program do not engage in savings and credit or other livelihoods activities, achieving impacts only through the BCC+ pathway. These programs often consist of awareness and behavior change promotion in groups facilitated by a trained community member, often using a participatory learning and action (PLA) methodology. A key distinction between BCC groups with and without a PLA approach is that women in groups using a PLA approach engage in a process of identifying shared problems, planning strategies, taking action collectively, and assessing impacts. This is different from traditional health education interventions that focus on transmission and learning of technical ‘messages’; PLA approaches aim to build community capacity to identify problems and act collectively to address them (Morrison et al., 2010).

Findings of studies evaluating different group-typologies and triggering different impact pathways were summarized to gain a general idea of their relative effectiveness (Table 6, Table 7). Studies evaluating microfinance and livelihoods SHG*-*based programs reported more frequently on anthropometric and dietary outcomes, while studies on BCC group-based programs were more likely to report on IYCF practices (Table 6). Among studies on microfinance and livelihoods SHG-based programs, 4 of 7 that measured anthropometric outcomes and 4 of 5 that measured dietary outcomes found positive and significant impacts on at least one outcome. Only one study measured the effect of these programs on IYCF practices and reported a null finding. However, 9 of 15 studies evaluating the impact of women's BCC group-based programs on IYCF practices reported positive and significant findings. In studies evaluating multi-sectoral group-based programs*,* positive and significant findings were reported for IYCF but not anthropometric outcomes. Less conclusive evidence exists across all program types on dietary outcomes.

**Table 6:** Findings on maternal and child nutrition outcomes by women's group intervention type and outcome category.

Intervention type and outcome category	Total studies (outcomes)	Pos./Sig. studies (outcomes)*	Neg./Sig. studies (outcomes)*	Null/Non-sig. studies (outcomes)*
**Microfinance group-based interventions**	5 (20)	3 (6)	0 (0)	5 (14)
Intake and diet	2 (2)	1 (1)	0 (0)	1 (1)
IYCF practices	0 (0)	–	–	–
Anthropometry	4 (18)	2 (5)	0 (0)	4 (13)
**Livelihoods self-help group-based interventions**	6 (71)	5 (10)	3 (8)	6 (53)
Intake and diet	3 (40)	3 (4)	1 (5)	2 (31)**
IYCF practices	1 (1)	0 (0)	0 (0)	1 (1)
Anthropometry	3 (30)	2 (6)	2 (3)	3 (21)***
**Multi-sectoral group-based interventions**	4 (9)	2 (3)	1 (1)	2 (5)
Intake and diet	0 (0)	–	–	–
IYCF practices	2 (3)	2 (3)	0 (0)	0 (0)
Anthropometry	2 (6)	0 (0)	1 (1)	2 (5)****
**BCC group-based interventions**	18 (59)	10 (20)	2 (2)	14 (37)
Intake and diet	3 (7)	0 (0)	1 (1)	2 (6)
IYCF practices	15 (35)	9 (19)	1 (1)	9 (15)
Anthropometry	3 (17)	1 (1)	0 (0)	3 (16)
**Multiple/unspecified group-based interventions**	3 (8)	1 (1)	0 (0)	3 (7)
Intake and diet	1 (2)	0 (0)	0 (0)	1 (2)
IYCF practices	1 (1)	1 (1)	0 (0)	0 (0)
Anthropometry	2 (5)	0 (0)	0 (0)	2 (5)

Numbers in parentheses indicate the number of times each outcome was reported, which may be more than once in a given study if results were reported separately for different subgroups.

*Alphabetized list of abbreviations*: BCC, behavior change communication; IYCF, infant and young child feeding.

* Significance considered at p < 0.05 level; positive implies a benefit of intervention while negative implies the opposite.

** 1 Additional outcome was positive-significant and 1 was negative significant at 10% (p< 0.1).

*** 3 Additional outcomes were positive-significant at 10% (p < 0.1).

**** 1 Additional outcome was positive-significant at 10% (p < 0.1).

**Table 7:** Findings on maternal and child nutrition outcomes by nutrition impact pathway triggered and outcome category.

Pathway triggered and outcome category	Total studies (Outcomes)	Pos./Sig. studies (outcomes)*	Neg./Sig. studies (outcomes)*	Null/Non-sig. studies (outcomes)*
**Income, Agriculture, Rights pathways**^a^	14 (98)	10 (19)	4 (9)	11 (70)
Intake and diet	4 (41)	4 (5)	1 (5)	2 (31)
IYCF practices	3 (4)	2 (3)	0 (0)	1 (1)
Anthropometry	8 (53)	4 (11)	3 (4)	8 (38)
**BCC+ pathway**^b^	22 (68)	12 (23)	3 (3)	16 (42)
Intake and diet	3 (7)	0 (0)	1 (1)	2 (6)
IYCF practices	17 (38)	11 (22)	1 (1)	9 (15)
Anthropometry	5 (23)	1 (1)	1 (1)	5 (21)

Numbers in parentheses indicate the number of times each outcome was reported, which may be more than once in a given study if results were reported separately for different subgroups.

*Alphabetized list of abbreviations*: BCC, behavior change communication; IYCF, infant and young child feeding.

* Significance considered at p < 0.05 level; positive implies a benefit of intervention while negative implies the opposite.

a The same set of studies triggered income, rights, and agriculture pathways (see Table 2), thus these pathways were combined to limit repetition of numbers in the table.

b In addition to behavior change communication, the intervention also included health and nutrition services including growth monitoring, supplementation, health checkups, and/or distribution of care packages.

When looking at outcomes by impact pathway triggered instead of group type, those that explicitly triggered the nutrition BCC+ pathway were most successful in improving maternal and child nutrition outcomes (Table 7). Ten of 18 studies reported at least one positive significant finding, and 19 (54%) of 35 IYCF outcomes were positively significantly related to being in the treatment group. There was positive but relatively weaker evidence for the impact of triggering income, agriculture and rights pathways on nutrition outcomes, with most studies triggering these pathways reporting mostly null effects.

## 6 Discussion

In this paper, we have proposed a conceptual framework to situate, examine, and interpret the findings of diverse studies on the impacts of group-based programs on nutritional outcomes. Our framework emphasizes the diversity of potential pathways to nutrition, emanating primarily from the group type, each of which triggers a different, yet interlinked, set of intermediate outcomes and pathways to impact. Our review of 36 eligible studies, that reported on a total of 167 different findings for nutrition outcomes, finds positive/significant, negative/significant, and non-significant findings for 40 (24%), 11 (7%), and 116 (69%) outcomes, respectively. Two studies added during the peer review process, although outside the period of the main search, do not skew our findings. Our assessment is that the predominance of null findings stem from a combination of program and/or implementation limitations, some of which we highlight below.

First, not all studies employed rigorous study designs; of 36 studies identified for review, only 12 were RCTs. This weakens the basis for conclusive evidence about the impact of group-based programs. Although conducting rigorous evaluations of complex programs is a challenge, strengthening the evidence base is imperative. Group-based development programs may take enormous efforts to implement, may impose opportunity cost burdens on participants, and may even cost more to establish and deliver than non-group-based programs. Despite potentially wide-ranging and highly cost-effective impacts, without a robust evidence-base that is built on high quality research, comparing these programs to other programs is not feasible.

Second, it is essential that research on the impacts of group-based programs on nutritional outcomes considers the potential pathways to impact, and assesses whether the program inputs delivered are adequate to trigger change in the desired outcomes in a given context. It is also crucial to invest in adequate measurement to help document intermediate outcomes along the impact pathway. This can ensure that interpretations of the impact, or lack thereof, and of links between women's group programs and nutritional outcomes are informed by robust contextual measurement of pathways.

Third, programs evaluated for nutrition impacts often lack components specifically oriented toward nutrition, or focus on only one pathway to nutrition impact. In some of these programs, other services (such as technology dissemination and credit) are “bundled” with group-based delivery, and the evaluation design did not have separate treatment arms for the modality of service delivery, making it impossible to disentangle the separate effects of group-based delivery. As with other research focusing on making development interventions more nutrition-sensitive (Ruel et al., 2013), programs with specific nutrition goals and linked activities such as nutrition behavior change are more likely to impact nutrition. Our review highlights that studies examining BCC group-based programs, which have an explicit nutrition goal and a focus on IYCF practices, often find impacts. Other programs, with more distal pathways to nutrition and a limited nutrition focus, were less likely to achieve nutrition impacts. Notably, behavior change groups addressing multiple nutritional behaviors and generating impacts on longer term outcomes such as anthropometry have not yet been deployed widely. Furthermore, few studies examine programs with a “multiple pathways to change” lens. This results in a very limited evidence base on programs that trigger all potential pathways, and only one study of those included in the review focused on a truly multisectoral set of pathways (Saha et al., 2015).

Fourth, few studies targeted nutritionally vulnerable age groups, which is central to achieving impact on nutritional goals such as child anthropometry. Although microfinance and livelihoods SHG-based programs have the capacity to generate long term impact because of access to and possible increased control of capital, these programs don’t explicitly target the crucial 1000-day window for nutrition impacts on children. However, findings in this paper on IYCF are promising and indicate that microfinance and livelihoods SHG-based programs may be able to deliver nutritional impacts by adding behavior change or other nutritional support components.

Finally, programs often lack the reach and duration necessary to achieve impact. These are central factors, not just for impact on nutrition but also on multiple outcomes. The literature reviewed in this paper also suggests that, across different types of group-based programs, adequate coverage as well as duration of exposure appear to be important. Several authors indicate that null findings are likely due to low coverage (Azad et al., 2010, More et al., 2012).

Our review itself is not without limitations, some of which derive from how the main outcome of interest for each reviewed study defines the reference point for process indicators. For example, where a primary outcome was neonatal mortality rate (Azad et al., 2010), prenatal care, delivery location, birth practices (e.g. cord care, safe delivery kit), and BF were considered as “process indicators” rather than outcomes. Thus, the set of outcomes and related intermediate measures varies by initial primary outcome for the studies. Our search strategy did not address this distinction between primary outcome and process indicators or intermediate outcomes. Second, we limited the search to articles that measured a specified set of nutrition outcomes, rather than studies that addressed intermediate outcomes in our conceptual framework. The latter approach would have expanded the scope of this review, but would have made it infeasible to conduct. Our primary goal was to identify research that had reported on nutritional outcomes as key end-points, and most articles reporting nutrition outcomes, particularly those from impact evaluations, tended to limit process indicators and focus on impact.

The evaluations that had significant and positive impacts, as well as those that did not, provide lessons for future nutrition programming in South Asia. A key insight is that the inclusion of explicit nutrition goals and actions in programs (as noted in Ruel and Alderman, 2013) made them more successful in delivering on improved nutrition outcomes. Another insight relates to targeting: one study found greater impacts on those with higher initial poverty (Deininger and Liu, 2013b), suggesting that there are gains to targeting nutrition interventions delivered through women's groups to the poorer groups in the population. Third, the role of collective action in strengthening impacts (or of poor collective action in the absence of impacts or even negative impact) (Bhutta et al., 2011, More et al., 2012) suggests that merely forming women's groups is not a panacea. The groups must effectively enable basic tenets of group-based engagement such as building social capital, promoting women's empowerment, and advocating to community leaders.

We acknowledge that our review does not represent all South Asian countries. This partially reflects the context in which group-based programs are being implemented, which may also be a function of the suitability of such programs in specific locations, as well as the need for more research along these dimensions in Bhutan, Afghanistan, Maldives and Sri Lanka. However, the search process did identify a range of group-based programs in different contexts, which suggests that the overall results may be applicable to other countries or contexts outside South Asia where similar conditions exist: resource scarcity, inequitable gender norms, and poor nutrition. Our findings are drawn from different contexts, even with a given country, and the results indicate that women's groups have potential for improving maternal and child nutrition outcomes. The evidence we reviewed helps us provide guidance on the components of such groups that may facilitate improved nutrition outcomes. Thus, our results should not be taken as prescriptive, even in the countries that are represented in this review. Finally, we note that for a program to be successful, it needs to be designed with both the context and the ultimate objective in mind and implemented well.

The complex nature of undernutrition in South Asia calls for multisectoral programs to tackle nutrition problems by addressing multiple determinants of undernutrition. Group-based programs, that engage women, have rich potential to trigger several pathways to change, and thus address several determinants through a single platform. However, the complexity and diversity of these programs implies that both implementers and evaluators should try to anticipate and then measure which pathways to impact are most likely to be successful in any given program context. Adequate investment into measurement of intermediate outcomes across potentially interconnected pathways is essential as many countries in South Asia embrace women's groups as a platform for social, behavioral and nutritional change.
